# Short-term and long-term success of electrical cardioversion in atrial fibrillation in managed care system

**DOI:** 10.1186/1755-7682-2-39

**Published:** 2009-12-12

**Authors:** Suman S Kuppahally, Elyse Foster, Stanford Shoor, Anthony E Steimle

**Affiliations:** 1 Division of Cardiology, University of Utah, 50 North Medical Drive, Salt Lake City, UT, 84132, USA; 2 Division of Cardiology, Department of Medicine, University of California, San Francisco, 505 Parnassus Ave, M314A San Francisco, CA, 94143-0214, USA; 3 Department of Medicine, Kaiser Permanente Santa Clara Medical Center, 710 Lawrence Expressway, Santa Clara, CA, 95051, USA.; 4 Department of Cardiology, Kaiser Permanente Santa Clara Medical Center, 710 Lawrence Expressway, Santa Clara, CA, 95051, USA

## Abstract

**Background:**

Initial success of electrical cardioversion (ECV) of atrial fibrillation (AF) has been reported in several studies as 50%-90%, of which only 50% patients remain in sinus rhythm (SR) at the end of one year. We conducted this study to see if outcomes of other trials are applicable in managed care setting.

**Methods:**

We conducted a retrospective study in 370 consecutive patients who underwent ECV for AF. They were reviewed for initial outcome of ECV and recurrence of AF after a successful ECV, with and without prophylactic antiarrhythmic drugs.

**Results:**

Initial success of ECV for AF was 65.7%. At one year, 47% remained in SR. AF for ≤ 3 months (p = 0.006) and pretreatment with antiarrhythmic drugs (p = 0.032) resulted in improved success. Predictors of recurrence were patients ≤ 65 years (p = 0.019), paroxysmal atrial fibrillation (PAF) (p = 0.0094) and alcohol consumption (p = 0.0074).

**Conclusion:**

Shorter duration of AF, prophylactic antiarrhythmic drugs and serial ECVs improve outcome of ECV in AF. For younger patients with PAF and alcohol consumption, due to higher recurrence of AF, rate control or ablative therapy may be the preferred strategy.

## Introduction

Atrial Fibrillation (AF) is the most common dysrhythmia encountered in clinical practice, accounting for approximately one third of hospitalizations for cardiac rhythm disturbance. Electrical cardioversion (ECV) is used to restore sinus rhythm (SR), both to alleviate associated symptoms and to prevent congestive heart failure and embolic complications.

The initial success of ECV has been reported as 50-90% in prior studies [1,2]. It is reported that younger patients with AF of less than 3 months duration are more likely to respond to ECV than the elderly patients with AF for > 36 months [2]. However, up to half of the patients are reported to have recurrence of AF within three to six months with the highest number of recurrences in the first 8 weeks [1,3,4]. Similar rates of recurrence have been reported in patients with paroxysmal AF (PAF) or atrial flutter [5]. Antiarrhythmic drugs have been used to improve the immediate and long-term success of cardioversion, but less favored due to unacceptable side effects [6-8].

We conducted this study to see if the results of the trials done at academic research centers with unrestricted availability of resources had similar outcome to that seen in a managed care setting where the practice is done in a cost-effective manner. This study was conducted to determine the short-term and long-term success of ECV in a large integrated managed care system. We also aimed to determine the predictors of initial success and recurrence of AF after successful ECV, including antiarrhythmic drugs.

## Methods

### Study Patients

All 734 ECVs performed at a Health Maintenance Organization (HMO), Kaiser Permanente Hospital, Santa Clara, California were identified from the hospital database by the ICD-9-CD code for ECV. Patients with AF by electrocardiogram or diagnostic code were then selected. Of these, medical records were available for retrospective review on 400 patients. Thirty patients were excluded because ECV was performed for ventricular tachycardia and ventricular fibrillation, resulting in a study population of 370 patients. An approval was obtained from the Kaiser Permanente Northern California Institutional Review Board (KPNC IRB). Anonymity of the patients was strictly maintained in study databases.

### Data Abstraction

Demographic information including age and gender, duration of AF and date of ECV; history of recent smoking and alcoholism were abstracted from the medical records. Medical history was available from both written medical records as well as from inpatient and outpatient electronic databases. Comorbidities were tabulated including: congestive heart failure, cardiomyopathy, valvular heart disease, chronic obstructive pulmonary disease, hyperthyroidism, diabetes mellitus, coronary artery disease and hypertension. Echocardiographic data, when available, were reviewed for left atrial (LA) dimension and left ventricular (LV) systolic function.

In the absence of comorbidities, the patient was diagnosed as lone AF. Patients were classified as having: paroxysmal or persistent AF as per American College of Cardiology/American Heart Association guidelines [9]. We pre-specified the following subgroups: Age ≤ 65 or > 65 years, male vs. female, duration of AF: 0-3 days, 4 days to 3 months, > 3 months. New onset AF was defined as 0-3 days.

### Protocol

Written informed consent was obtained before the ECV from each patient. Generally ECV was performed with pacing pads placed anteriorly over the chest and posteriorly in the interscapular area, using single direction wave shock with escalating energy levels (50-360 Joules) at the discretion of the cardiologist. Patients who underwent non-emergent ECV were anticoagulated with warfarin for at least 4 weeks prior to the procedure and had therapeutic INR at the time of ECV or underwent transesophageal echocardiogram to rule out intracardiac thrombus. In case of emergent ECV, intravenous heparin was administered.

At the time of ECV, if the patient was taking an antiarrhythmic drug, it was recorded as ECV on antiarrhythmic pretreatment. The ECV was defined as successful if the patients were in SR immediately after the procedure. Patients who underwent second ECV with or without pretreatment with antiarrhythmic drugs were recorded. All the charts were reviewed in the year post-cardioversion for recurrence of AF including outpatient visits, emergency room visits or rehospitalization.

### End Points

The primary end point of the study was immediate success of ECV. The secondary end point was defined as the first recurrence of AF after successful ECV.

### Statistical Analysis

We conducted both univariate and multivariate analyses. For the univariate analysis, we used Chi-square tests to analyze the relationship between the variables as shown in table 1 with the success/failure of ECV. For the multivariate analysis, logistic regression was used and adjusted for all the variables. P-value of < 0.05 was considered statistically significant.

**Table 1:** Characteristics of patients who underwent electrical cardioversion for atrial fibrillation

Total number of patients	370
Age of patients (mean ± SD years)	67 +/- 12

≤ 65 years	137

>65 years	233

Men/Women	239/131

**Underlying coexisting diseases (number of patients)**	

Coronary artery disease	150

Congestive heart failure	116

Mitral stenosis	16

Mitral regurgitation	92

Paroxysmal atrial fibrillation	196

Hypertension	185

Cardiomyopathy	75

Diabetes mellitus	46

Chronic obstructive pulmonary disease	51

Alcoholism	105

Smoking	96

Lone atrial fibrillation	27

Duration of atrial fibrillation	

0-3 days	91

4 days-3 months	105

>3 months	174

Left atrial size (157 patients)	

≤ 4.2 cm	72

>4.2 cm	85

Left Ventricular Ejection Fraction	

Normal	129

Reduced	40

## Results

**Figure 1:**
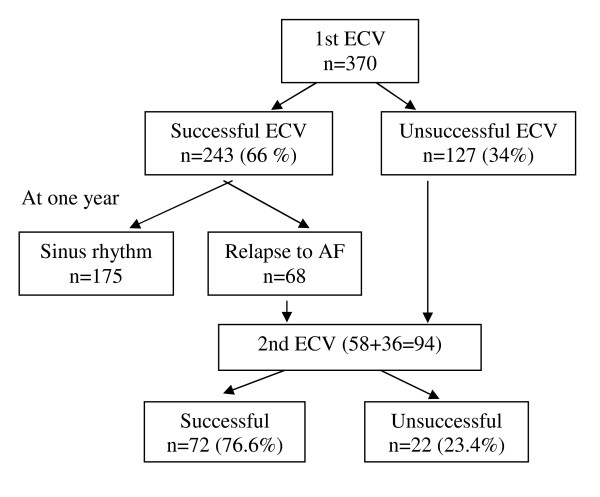
**Results of serial electrical cardioversion in atrial fibrillation and follow-up over one year**. (ECV-Electrical cardioversion, AF-atrial fibrillation).

Characteristics of patients are presented in table 1. Twenty-seven patients (7.3%) had lone AF. The mean age was 67 +/- 12 years. Of the 370 initial attempts at cardioversion, 243/370 (65.7%) patients were successful. At the end of one year, 175 patients remained in SR (72% of those whose initial cardioversion was successful and 47% overall) and 53% of the total were in AF. Of the 68 patients who relapsed into AF by one year, 58 patients underwent repeat ECV. Thirty-six patients (36/127) whose 1st cardioversion failed also underwent repeat ECV, for a total of 94 patients undergoing 2nd ECV (Figure 1). Overall, 66/94 patients were on antiarrhythmic drugs at the time of 2nd ECV. With the second attempt of ECV, 72/94 (76.6%) patients were converted to SR. Patients had a more favorable outcome with the 2nd ECV as compared to initial ECV (76.6% vs 62.67%, p = 0.042).

### Effect of Antiarrhythmics

**Figure 2:**
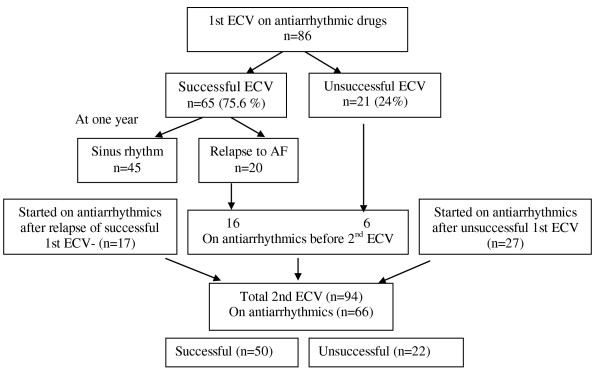
**Results of serial electrical cardioversion in atrial fibrillation in patients after prophylactic antiarrhythmic drugs and follow-up over one year**. (ECV-Electrical cardioversion, AF- atrial fibrillation).

Antiarrhythmic drugs included procainamide, amiodarone, quinidine, ibutilide, propafenone, sotalol or flecainide according the preference of the treating physician (table 2). Eighty-six patients (86/370) were receiving antiarrhythmic drugs at the time of 1st ECV. Of these, more patients (n = 65) on antiarrhythmic drugs successfully converted to normal SR after ECV compared to those not receiving (n = 178) antiarrhythmic drugs (75.6% vs. 62.7%, p = 0.027). Using logistic regression to adjust for all the variables in table 1, antiarrhythmic use remained independently associated with successful cardioversion (p = 0.032, Table 3). Of the 94 patients undergoing 2nd ECV, 66 were receiving antiarrhythmic drugs (figure 2). There was no significant difference in the success of 2nd ECV between those receiving and not receiving antiarrhythmic drugs (75.7% vs 78.6%, p = 0.768). Antiarrhythmic drugs did not decrease the incidence of recurrence of AF after a successful ECV (45/65, 69% vs. 130/178, 73%, p = NS). Two patients on antiarrhythmics had spontaneous conversion to SR after failed initial ECV.

**Table 2:** Outcome of electrical cardioversion in atrial fibrillation on prophylactic antiarrhythmic treatment before the 1^st ^and 2^nd ^electrical cardioversion

Antiarrhythmic drug	Patients with successful 1^st ^ECV (%)	Patients with successful 2^nd ^ECV (%)
Procainamide	22 (73)	5 (71)

Amiodarone	15 (75)	17 (59)

Quinidine	9 (75)	2 (100)

Ibutilide	6 (75)	4 (100)

Propafenone	6 (86)	3 (100)

Sotalol	6 (86)	17 (94)

Flecainide	1 (50)	2 (67)

No drug	178 (63)	22 (79)

**Table 3:** Co-morbidities affecting success of initial electrical cardioversion (logistic regression)

Parameter	p-value	Odds Ratio Estimates (95%) Confidence Intervals	
Age: ≤ 65 and > 65 years	0.532	0.519	1.404
Sex: Male vs. Female	0.086	0.939	2.578
Duration: ≤ 3 m vs. > 3 months	0.006	0.332	0.837
Duration: 0-3 days vs. > 3 months	0.003	1.361	4.693
Duration: 4 days-3 m vs. > 3 months	0.113	0.902	2.655
On antiarrhythmic medications	0.032	1.056	3.420
Lone atrial fibrillation	0.736	0.325	2.215
Congestive heart failure	0.406	0.437	1.398
Cardiomyopathy	0.968	0.531	1.934
Mitral stenosis	0.517	0.393	6.390
Mitral regurgitation	0.684	0.612	2.111
Pulmonary Disease	0.533	0.372	1.669
Diabetes mellitus	0.070	0.942	4.525
Coronary artery disease	0.414	0.744	2.049
Hypertension	0.867	0.592	1.556
Paroxysmal atrial fibrillation	0.498	0.726	1.932
Alcoholism	0.173	0.410	1.175
Smoking	0.682	0.617	0.092

### Predictors of Short-term and Long-term Success of ECV in AF

#### Age and Sex

The success rate was similar in younger and older patients after the 1^st ^ECV (≤ 65 years: 65.7% vs >65 years: 65.7%). However, a greater number of younger patients relapsed in to AF during the course of one year (≤ 65 years: 35% vs >65 years: 22%, p = 0.009 by univariate analysis). Younger patients were twice as likely to have recurrence of AF even after adjusting to gender, duration of AF and comorbid conditions (p = 0.019, OR = 2.24, 95% confidence intervals, table 4).

**Table 4:** Co-morbidities affecting recurrence of atrial fibrillation after a successful electrical cardioversion (logistic regression)

Parameter	p-value	Odds Ratio Estimates (95%) Confidence Intervals	
Age: ≤ 65 vs. > 65 years	0.019	1.136	4.424
Sex: Male vs. Female	0.815	0.536	2.205
Duration: 0-3 days vs. > 3 months	0.607	0.380	1.759
Duration: 4 days-3 m vs. > 3 months	0.692	0.539	2.535
On antiarrhythmic medication	0.948	0.475	2.006
Lone atrial fibrillation	0.405	0.089	2.661
Congestive heart failure	0.884	0.413	2.142
Cardiomyopathy	0.885	0.373	2.343
Mitral stenosis	0.422	0.429	7.533
Mitral regurgitation	0.293	0.277	1.473
Pulmonary Disease	0.540	0.209	2.270
Diabetes mellitus	0.061	0.124	1.048
Coronary artery disease	0.760	0.570	2.158
Hypertension	0.904	0.500	1.848
Paroxysmal atrial fibrillation	0.009	1.257	5.149
Alcoholism	0.007	1.306	5.627
Smoking	0.099	0.183	1.157

By univariate analysis, females had greater success with initial ECV than males (Females: 72.52% vs Males: 62%, p = 0.04). But, when adjusted for comorbid conditions, sex did not did not affect the success of ECV or relapse of AF.

#### Co-morbid Conditions

None of the coexisting medical problems significantly influenced the immediate outcome of initial ECV or repeat ECV for recurrent AF.

Patients with PAF (p = 0.009) and history of alcohol consumption (p = 0.007) had a significantly higher likelihood of recurrence of AF within one year after successful ECV (Table 4).

#### Duration of AF

**Figure 3:**
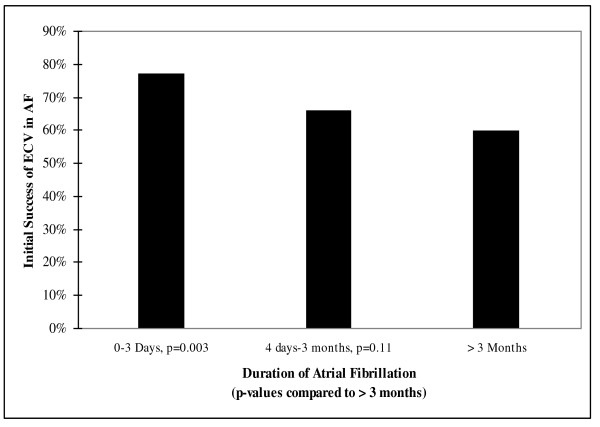
**Effect of duration on the success of electrical cardioversion in atrial fibrillation**.

Duration of AF was inversely related to initial success rate (0-3 days: 76.9%, 4 days-3 months: 65.7%, > 3 months: 59.8%), (Figure 3). The difference between 0-3 days and > 3 months was significant (p = 0.003) while the difference between 4 days-3 months and > 3 months was not (p = 0.11). Recurrence of AF in these three groups after successful ECV was not affected by duration of AF.

#### Echocardiographic Parameters

Echocardiography reports were available on 169 patients. LA dimension was reported in 157 patients that ranged from 3.2 cm to 7.0 cm. In our observation, LA size did not influence the short or long term success of ECV (≤ 4.2 cm: 71.8% vs. >4.2 cm: 61.6%, p = 0.10). No significant difference was noted when different cut-off values (LA size of 4.6 cm and 5.0 cm) were analyzed. LV systolic function was reported as normal in 129 patients and reduced in 40 patients; LV dysfunction did not correlate with success of initial ECV.

In the cohort of patients with known LA size (157 patients), after adding the LA size to the other parameters in logistic regression analysis, the positive predictors of success including shorter duration of AF and prophylactic antiarrhythmic drugs seen in the entire group was not observed. However, patients with a history of alcohol consumption had lower success of ECV if LA size was > 4.2 cm (p = 0.04). Younger patients had more recurrences in this group as well (p = 0.007).

## Discussion

This is the largest study involving consecutive patients undergoing ECV within an integrated medical delivery system. The extensive database available within this HMO provided unique clinical information since most patients used this facility exclusively for their health care.

In this population, overall success of ECV in restoring AF to SR at our facility was 67%, which falls in the wide range reported in the literature [1,2]. Shorter duration of AF of 0-3 days was a significant determinant of successful ECV, which is in accordance to prior trials [2,10]. Interestingly, the long-term recurrence of AF after ECV was not affected by the duration of AF in our study. In contrast, Frick et al followed the patients for short-term of one month after ECV and reported that a duration of AF < 6 months predicted success [11]. In another study, duration of AF < 1 year predicted success [12]. Based on the variable results, it is reasonable to attempt ECV in patients with AF even in those with unknown duration of AF. Besides duration of AF, pretreatment with antiarrhythmic drugs predicted successful ECV.The benefit of antiarrhythmic drugs prior to cardioversion for initial restoration of sinus rhythm was similar to several prospective trials [7,13-15], supporting their use when possible. This observed benefit needs to be weighed against the potential adverse side effects of these agents and the potential delay in offering cardioversion. But unlike other studies [8,16], antiarrhythmic drugs did not reduce relapses significantly in our study which could be limited by small sample size and unavailable information about the duration of therapy and withdrawals.

In the follow-up over one year, 72% of the patients who were successfully cardioverted to normal SR remained in SR. Overall, 53% of the study patients were in AF at one uear follow-up, as compared to 46% patients noted in AFFIRM trial [17]. Several parameters were found to affect the recurrence of AF after a successful ECV, including age younger than or equal to 65 years, history of PAF and alcohol consumption. The higher recurrence of AF in younger patients after a successful ECV is contrary to other reports [1,2], possibly due to the younger patients in our study having higher rates of alcohol consumption and PAF. This is an important observation for younger patients who may have limitations in activities and are reluctant to take drugs as they may be better served with ablative therapies. In the Framingham study, the authors observed little association between moderate alcohol consumption and the risk of AF, but a significantly increased risk of AF with a consumption of > 36 g/day or about 3 drinks/day [18]. It also brings up an important question whether some patients were actually not appropriate candidates for ECV, such as those with significant triggering factors like alcohol leading to recurrence. Rate control strategy may be preferable in such patients.

A high rate of successful repeat ECV in patients with failed initial ECV or relapse after a successful initial ECV was noted. Van Gelder et al have reported that serial ECV may be beneficial in younger patients with fair exercise tolerance and with shorter than 36 months duration of AF [2].

In our study, patient's age did not affect the success/failure rate of initial ECV, which is accordance with the results of prior studies [10,19]. LA dimension and LV systolic function also did not predict successful ECV in AF. Although this finding may have been affected by the availability of this echocardiographic data in only a subset or our patients, prior studies have not consistently shown an effect of LA dimension and LV function on success of ECV [20,21]. In clinical practice, however, ECV is commonly offered for AF to patients with large atria and reduced LV function. Larger LA does not preclude a favorable outcome of ECV in AF and results in reversal of atrial size [22]

Rate vs rhythm control strategies have been studied by large prospective trials, and have concluded that the two approaches are associated with similar morbidity, cardiovascular events, stroke and quality of life [23,24]. All of the patients in the RACE trial had a minimum of one prior cardioversion. The majority of patients in the AFFIRM trial had also been previously cardioverted. In a subset of patients with pre-existing heart failure (23% of the population), rhythm control actually seemed to be favorable. Rhythm control strategy may be the preferred treatment of choice for patients with disabling symptoms due to AF. Loss of atrial contribution to ventricular filling can lead to hemodynamic deterioration in patients with preload dependent lesions such as aortic stenosis and diastolic heart failure. In elderly patients with persistent arrhythmia, cognitive function may be impaired as compared with age-matched controls in sinus rhythm [25], probably due to chronic hypoperfusion or cerebral microembolism.

Persistent AF over long duration is known to cause electrophysiological, anatomic, and pathological changes, with decreased atrial refractoriness [26,27]. This results in higher incidence of failure of ECV in patients with longer duration of AF (> 3 months) as seen in our study as well as in prior trials [2,10], which reinforces the need to attempt cardioversion, if planned, as early as possible. Even intermittent AF can lead to atrial fibrosis increasing the risk of persistent AF [28]. Radiofrequency ablation and pulmonary vein (PV) isolation are evolving techniques in patients with refractory AF. PV isolation is particularly beneficial in PAF, which have shown to reduce the recurrences of AF and improve the quality of life even after recurrences [29-31]. Khargi et al showed better success rate after Cox-Maze III procedure in younger patients with PAF [32].

### Limitations

There are several limitations to our study related to its retrospective, nonrandomized study design. Although our registry provided extensive, reliable data, there were missing data points. The history of alcohol intake by the subjects cannot be clarified to know the amount and frequency of alcohol consumption. Neither the duration or doses of antiarrhythmic drugs nor any proarrhythmic events were recorded. Another limitation is that recurrence of AF is based on intermittent and inconsistent monitoring. Studies with continuous monitoring with an implantable monitoring device have demonstrated higher rates of recurrence [33].

## Conclusions

In the managed care setting, the success of ECV is comparable to the results of other centers, with two thirds of patients showing immediate success of ESV and about 50% patients remaining in SR at the end of one year. Patient's age may not predict the initial success of electrical cardioversion. A longer duration of AF (>3 months) can be associated with lower success rate of ECV and serial ECV may improve the outcome. Higher relapses of AF after successful ECV in younger patients with PAF and history of alcohol consumption suggests that rate control or ablative therapy may be the preferred strategy for these patients. Antiarrhythmic drugs can improve the outcome of ECV, but their use requires careful weighing of potential risks and benefits.

## Competing interests

The authors declare that they have no competing interests.

## Authors' contributions

SK contributed to the conception and design of the study, acquisition of data, analysis and interpretation of data, and writing the manuscript. EF contributed to the interpretation of data and writing the manuscript. SS contributed to the design of the study, interpretation of the data, coordination of the project and writing the manuscript. AS contributed to the design of the study, interpretation of data and writing the manuscript. All authors read and approved the final manuscript.
